# Intratumoral immune heterogeneity of prostate cancer characterized by typing and hub genes

**DOI:** 10.1111/jcmm.17641

**Published:** 2022-12-16

**Authors:** Jianpeng Han, Yan Zhou, Chundong Zhang, Jianyong Feng, Junhao Wang, Kuo Guo, Wenbin Chen, Yongzhang Li

**Affiliations:** ^1^ Department of Urology Hebei Provincial Hospital of Traditional Chinese Medicine Shijiazhuang China; ^2^ Department of Function Hebei Provincial Hospital of Traditional Chinese Medicine Shijiazhuang China

**Keywords:** hormone therapy, hub gene, immune cell, prognosis

## Abstract

Discordant abundances of different immune cell subtypes is regarded to be an essential feature of tumour tissue. Direct studies in Prostate cancer (PC) of intratumoral immune heterogeneity characterized by immune cell subtype, are still lacking. Using the single sample gene set enrichment analysis (ssGSEA) algorithm, the abundance of 28 immune cells infiltration (ICI) were determined for PC. A NMF was performed to determine tumour‐sample clustering based on the abundance of ICI and PFS information. Hub genes of clusters were identified via weighted gene co‐expression network analysis (WGCNA). The multivariate dimensionality reduction analysis of hub genes expression matrix was carried out via principal component analysis (PCA) to obtain immune score (IS). We analysed the correlation between clustering, IS and clinical phenotype. We divided the 495 patients into clusterA (*n* = 193) and clusterB (*n* = 302) on the basis of ICI and PFS via NMF. The progression‐free survival (PFS) were better for clusterA than for clusterB (*p* < 0.001). Each immune cell subtypes was more abundant in clusterA than in clusterB (*p* < 0.001). The expression levels of CTAL‐4 and PD‐L1 were lower in clusterB than in clusterA (*p* < 0.001 and *p* = 0.006). We obtained 103 hub genes via WGCNA. In the training and validation cohorts, the prognosis of high IS group was worse than that of the low IS group (*p* < 0.05). IS had good predictive effect on 5‐year PFS. The expression of immune checkpoint genes was higher in the low IS group than in the high IS group (*p* < 0.01). Patients with low IS and receiving hormone therapy had better prognosis than other groups. The combination of IS and clinical characteristics including lymph node metastasis and gleason score can better differentiate patient outcomes than using it alone. IS was a practical algorithm to predict the prognosis of patients. Advanced PC patients with low IS may be more sensitive to hormone therapy. CXCL10, CXCL5, MMP1, CXCL12, CXCL11, CXCL2, STAT1, IL‐6 and TLR2 were hub genes, which may drive the homing of immune cells in tumours and promote immune cell differentiation.

## 1 INTRODUCTION

The incidence and mortality of prostate cancer (PC) ranked second and fifth in the spectrum of male malignant tumour diseases worldwide.^1^, ^2^ With the ageing of population and the change in lifestyle, both the incidence and mortality of PC have been increasing year by year.^3^, ^4^ Prostate cancer is a medical problem that affects more than 10 million older men.^1^ About 1.3 million new cases of prostate cancer are diagnosed worldwide each year.^1^ Seven per cent of all men with prostate cancer are at risk for metastasis.^1^, ^2^ Metastatic prostate cancer significantly increases the risk of death.^1^, ^3^ More than 400,000 men die each year from metastatic prostate cancer.^1^ This figure shows an increasing trend. By 2040, that number could double to 800,000.^1^

Although the diagnosis and treatment level of PC has made remarkable progress, due to the insidious onset of prostate cancer, most of the patients with prostate cancer have been initially diagnosed at advanced stage with poor prognosis.^5^, ^6^ Treatment strategies for PC are complex and require long‐term follow‐up and high cost.^1^, ^7^, ^8^, ^9^ Moreover, the current treatment options for advanced PC are limited without good treatment effects and with high rates of recurrence and drug resistance.^7^, ^9^

Immunotherapy has been applied for some tumour types, including prostate, lung, bladder and breast cancers, offering increased safety and fewer adverse events and provides new insights into oncotherapy, especially for advanced tumours.^10^, ^11^, ^12^, ^13^ Therapies that target immune checkpoints appear to have huge therapeutic potential, with wide recognition of cytotoxic T lymphocyte‐associated antigen 4 (CTLA‐4) and programmed cell death ligand 1 (PD‐L1).^14^, ^15^ At present, there are about 146 kinds of immune checkpoint inhibitors discovered and applied in clinical treatment, which have changed the treatment mode of many solid tumours, benefiting tumour patients and improving prognosis. However, 50%–60% of patients with cancer do not consistently respond to immunotherapy and have a poor prognosis.^16^, ^17^, ^18^ Unselected prostate cancer patients did not benefit from immunotherapy targeting immune checkpoints as expected. Therefore, it is vital to illuminate the landscape of immune cells infiltration (ICI) in tumour tissue in order to characterize intratumour immune heterogeneity, evaluate the sensitivity of patients to immunotherapy and determine the prognosis of patients.

Most previous studies have focused on the influence of several distinct types of immune cells on PC.^19^, ^20^, ^21^ Immunosuppression, as reflected by a low absolute lymphocyte count, defective natural killer (NK) cell activity or antigen presentation disorder, has been proposed to lead to the occurrence and progression of PC.^22^, ^23^, ^24^, ^25^ In addition, immunosuppressive cells and cytokines in the tumour microenvironment have been shown to contribute to immune escape in PC.^26^, ^27^ However, direct studies of intratumoral immune heterogeneity characterized by immune cell subtype, are still lacking.

Therefore, our study designed to illustrate the intratumoral immune heterogeneity of ICI in PC using non‐negative matrix factorization (NMF). Correlations between the clustering classification and prognosis, and CTLA‐4 and PD‐L1 expression levels were explored. We identified hub genes between clusters, which may drive immune cell homing and differentiation. Based on principal component analysis (PCA) of hub genes expression, we proposed an immune score (IS) calculation method that can be applied for prognosis and immunotherapy sensitivity assessment and screening of hormone therapy‐sensitive population. Our study may provide clues to the mechanism of immune cell enrichment in tumours.

## 2 MATERIALS AND METHODS

### 2.1 Data download

RNASeqV2 normalized data files for PC were downloaded from The Cancer Genome Atlas (TCGA), which contains 495 tumour samples. RNA expression profiles were summarized, log‐transformed and combined into a matrix. In addition, the corresponding clinical data files (*n* = 495) were downloaded. We sorted out clinical data files and extracted progression‐free survival (PFS) times and statuses. Clinical characteristics, including age, sex, TNM stage and history of treatments, were extracted and collated. Furthermore, we downloaded data from the gene expression omnibus (GEO) database (GSE51066 and GSE70769) to perform external validation, including 85 and 94 PC patients, respectively. The downloaded data are publicly available and open‐ended; therefore, approval by a local ethics committee was not required.

### 2.2 Evaluation of ICI and clustering via NMF

Using the single sample gene set enrichment analysis (ssGSEA) algorithm, the abundance of 28 ICI was determined for PC. Correlations between the 28 immune cell subtypes were explored using the spearman rank correlation Test. A NMF was performed to determine tumour‐sample clustering based on the abundance of ICI and PFS information. To further study the correlations between clustering and clinical characteristics, survival analyses with PFS was performed for different clusters using Kaplan–Meier curves. A Wilcoxon test was performed to compare differences in the expression of immune checkpoint and the abundance of ICI between different clusters.

### 2.3 Identification of hub genes

Hub genes of clusters were identified via weighted gene co‐expression network analysis (WGCNA). The co‐expression weighted network of all genes was constructed using R software and R package (WGCNA). The correlation between modules and clinical features was calculated. The screening criteria for hub genes were module membership (MM) > 0.8 and gene significance (GS) > 0.5. We constructed protein–protein interaction (PPI) network of hub genes based on String database. Hub genes of PPI network were identified by Cytoscape software (V3.7.1) and cytoHubba plug‐in.

### 2.4 Calculation of IS

The multivariate dimensionality reduction analysis of hub genes expression matrix was carried out via principal component analysis (PCA) to obtain IS. Immune score = ∑PC1(B+)–∑PC1(B−). PC1(B+) represented the first principal component of the hub genes in the modules positively correlated with clusterB. PC1(B−) represented the first principal component of the hub genes in the modules negatively correlated with clusterB. According to the optimal cut‐off value of IS, patients were divided into high and low IS groups. We explored the relationship between IS and clinical features and prognosis. Receiver operating characteristic (ROC) curve was applied to test the predictive efficacy of IS for 5‐year PFS.

### 2.5 Statistical analysis

All analyses were conducted using R software version 3.3. All statistical tests were two‐sided, and *p*‐values of <0.05 were considered statistically significant.

## 3 RESULTS

### 3.1 NMF clustering on the basis of ICI and PFS

**FIGURE 1:**
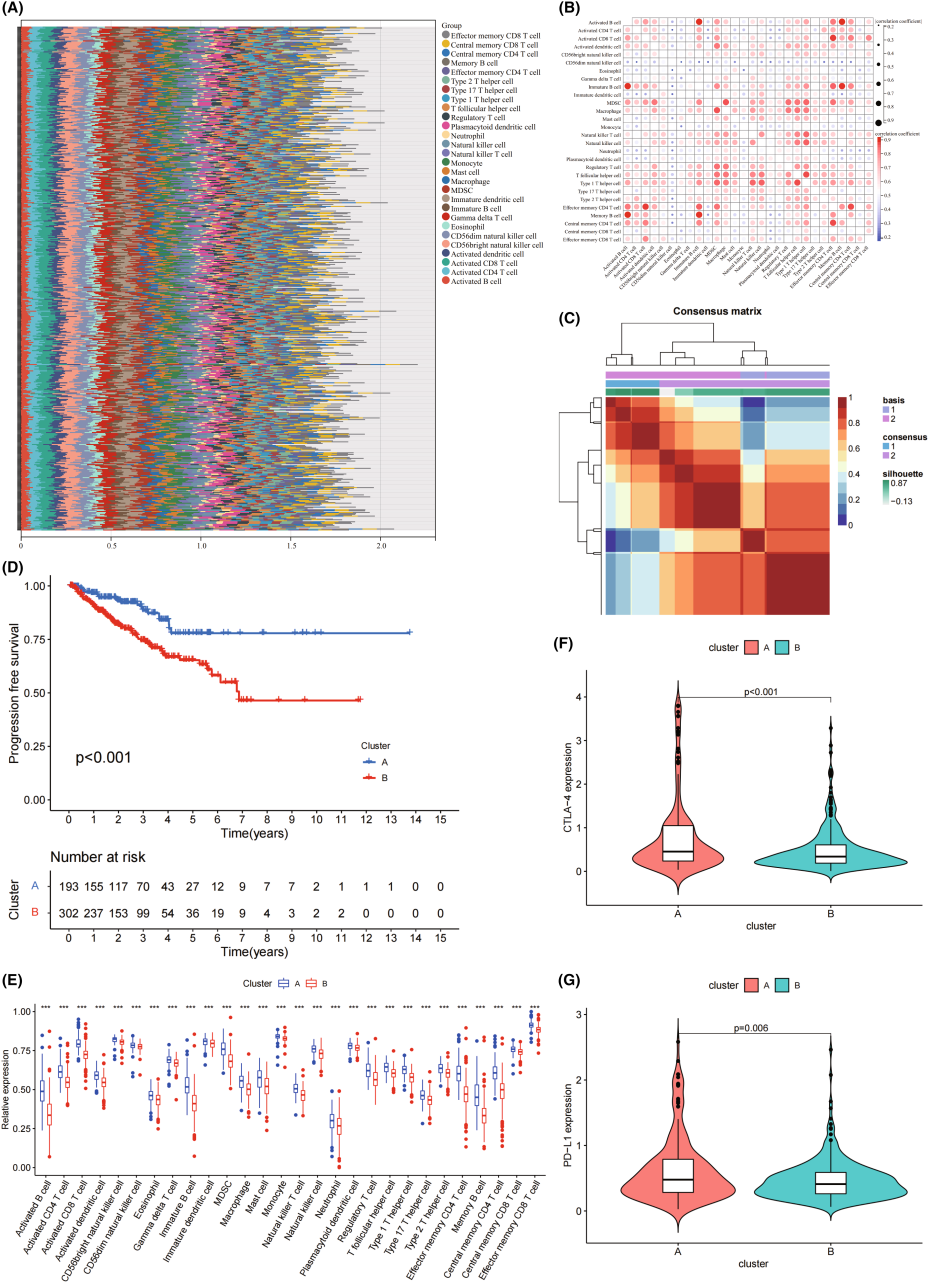
NMF clustering on the basis of ICI and PFS. (A) A bar plot shows the proportions of 28 immune cell subtypes in each sample (*n* = 495). (B) A correlation matrix for all 28 immune cell subtypes. (C) A NMF clustering matrix shows clusterA (*n* = 193) and clusterB (*n* = 302). (D) PFS comparison between clusterA and clusterB. (E) Differences of immune cell subtypes between clusterA and clusterB. (F and G) Differences of CTLA‐4 and PD‐L1 between clusterA and clusterB. NMF, non‐negative matrix factorization; PFS, progression‐free survival; ***p < 0.001

The ICI abundance of each PC sample (*n* = 495) was displayed in Figure 1A. The interactions between different immune cell subtypes in PC were positive. (Figure 1B). We divided the 495 PC patients into clusterA (*n* = 193) and clusterB (*n* = 302) on the basis of ICI and PFS via NMF (Figure 1C). These two clusters showed good intergroup differentiation. The progression‐free survival (PFS) (*p* < 0.001) were better for clusterA than for clusterB (Figure 1D). Each immune cell subtypes was significantly more abundant in clusterA than in clusterB (*p* < 0.001, Figure 1E). The expression levels of CTLA‐4 and PD‐L1 were lower in clusterB than in clusterA (*p* < 0.001 and *p* = 0.006, Figure 1F,G).

### 3.2 Screening of hub genes between clusters

**FIGURE 2:**
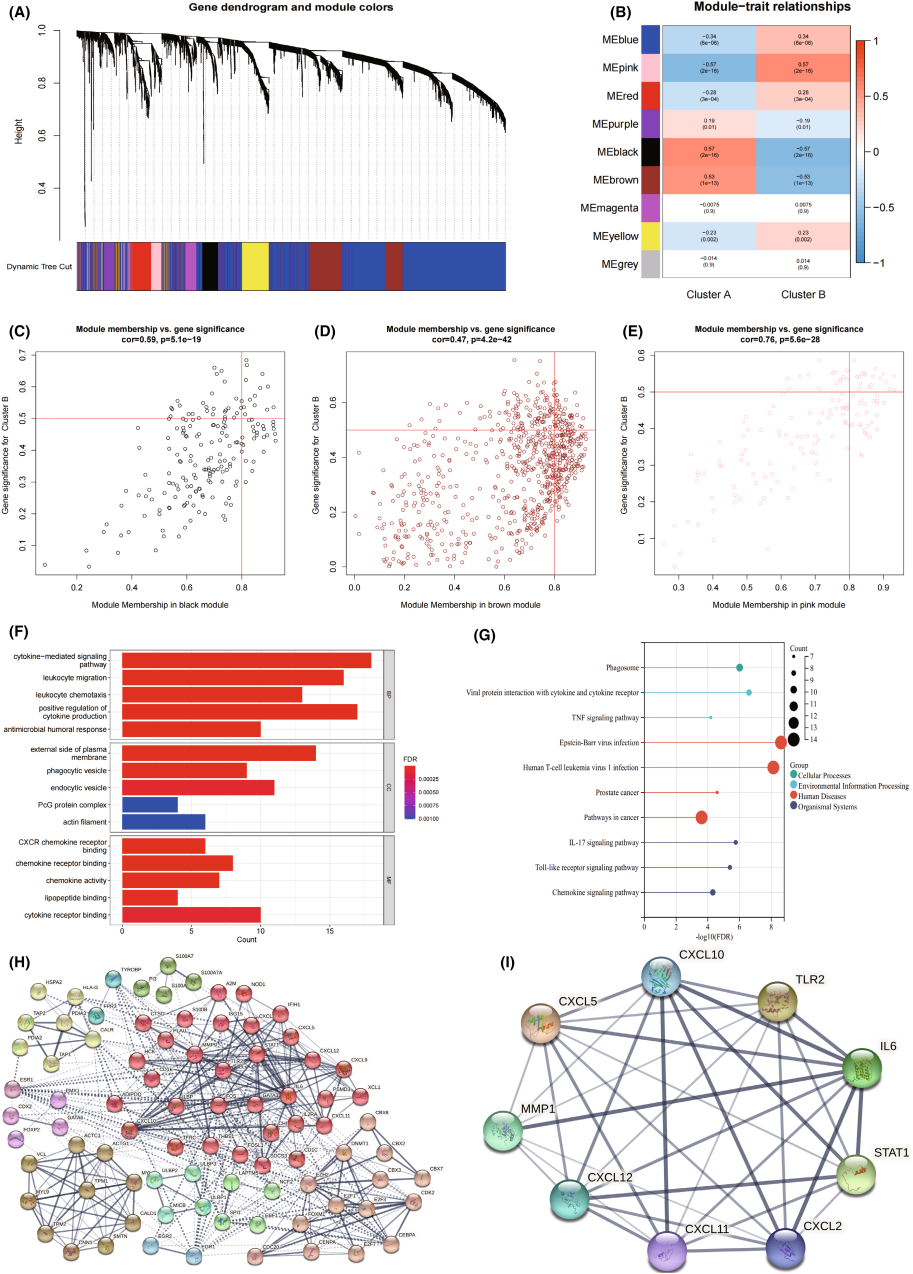
Key genes screening by WGCNA. (A) Co‐expression module construction by WGCNA. The tree branches represent genes, and the colours represent co‐expression modules. (B) Correlation between module genes and clusters. Red represents positively correlated with samples, blue represents negatively correlated with samples. The number at the top of the square represents the correlation coefficient, and the number in parentheses represents the *p*‐value. (C–E) Key genes screening by MM and GS in black, brown and pink modules. (F) GO enrichment analysis of key genes. (G) KEGG enrichment analysis of Key genes. (H) PPI network of key genes in the form of MCL. The colours represent different categories, the solid lines represent associations between genes within the same category, and the dashed lines represent associations between genes in different categories. (I) Hub gene network. GS, gene significance; MCL, Markov clustering; MM, module membership; PPI, protein‐–protein interaction network; WGCNA, weighted gene co‐expression network analysis

Gene co‐expression networks were constructed by WGCNA. Genes were enriched in 9 different modules (Figure 2A). Brown module (*r* = −0.53, *p* = 1e−13) and black module (*r* = −0.57, *p* = 2e−16) were negatively correlated with clusterB, whilst pink module (*r* = 0.57, *p* = 2e−16) was positively correlated with clusterB (Figure 2B). With MM > 0.8 and GS > 0.5 as screening criteria, we obtained 103 hub genes in black (*n* = 17), brown (*n* = 65) and pink modules (*n* = 21) (Figure 2C–E, Table S1). These genes were significantly enriched in immune‐related GO items and KEGG pathways (Figure 2F,G). PPI network of hub genes in the form of MCL was shown in Figure 2H. C‐X‐C ligand (CXCL) 10, CXCL5, matrix metalloproteinase 1 (MMP), CXCL12, CXCL11, CXCL2, recombinant signal transducer and activator of transcription 1 (STAT1), Interleukin‐ 6 (IL‐6) and toll‐like receptor 2 (TLR2) were hub genes for PPI network (Figure 2I).

### 3.3 Correlation between IS and PFS

**FIGURE 3:**
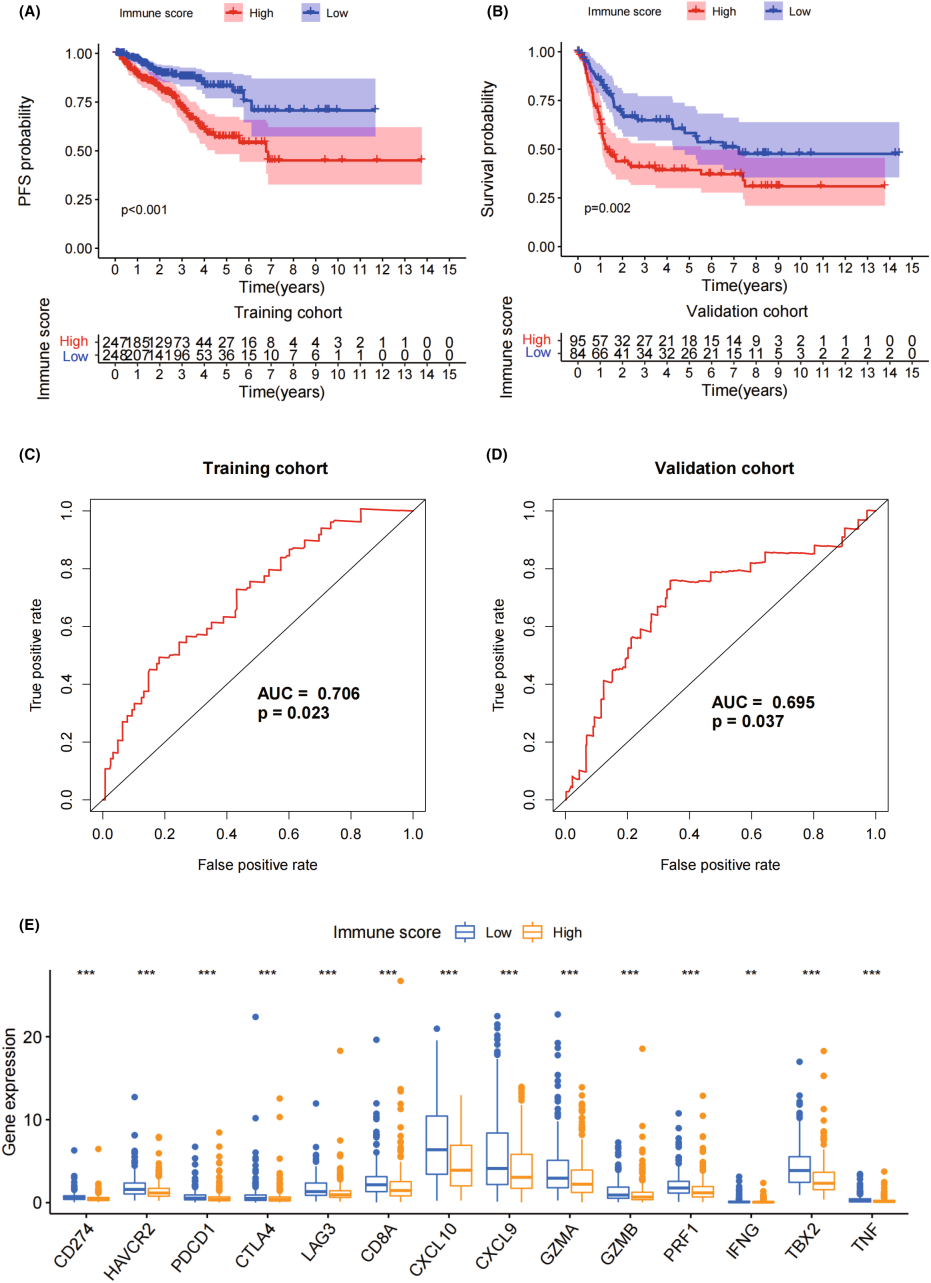
Immune score and PFS. (A and B) PFS analysis between high and low immune score group in training cohort and validation cohort. Different colour shadows indicate confidence intervals. (C and D) ROC curve of immune score predicting 5‐year PFS in training cohort and validation. (E) Expression level of immune checkpoint genes between high and low immune score group. AUC, area under curve; PFS, progression‐free survival; ROC, receiver operating characteristic; **p < 0.01; ***p < 0.001

The IS for PC patients was obtained by dimensionality reduction of the expression levels of 103 hub genes by PCA. The PC patients whose IS was greater than the optimal cut‐off value were assigned to the high IS group, otherwise, they were assigned to the low immune score group. In the training cohort of the PC patients from TCGA database, the prognosis of high IS group was worse than that of the low IS group (*p* < 0.001, Figure 3A). A similar trend was observed in the validation cohort of the PC patients from GEO database (*p* = 0.002, Figure 3B). In the training and validation cohort, IS had good predictive effect on 5‐year PFS (Figure 3C,D). The expression of immune checkpoint genes was higher in the low IS group than in the high IS group in the PC patients (*p* < 0.01, Figure 3E).

### 3.4 IS and clinical characteristics

**FIGURE 4:**
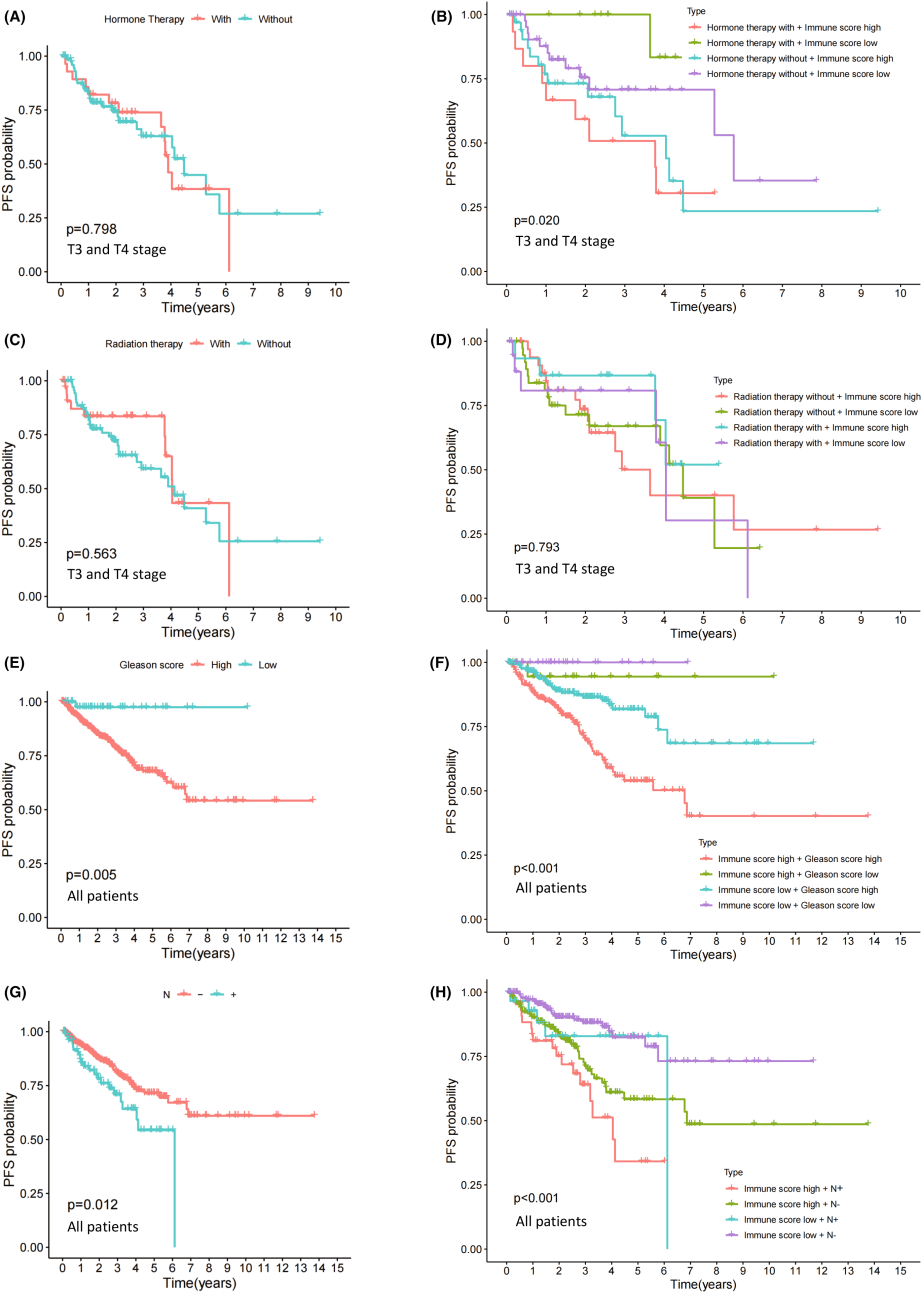
Immune score and clinical characteristics. (A) PFS differences of stage T3 and T4 patients with and without hormone therapy. (B) PFS differences of patients classified by hormone therapy combined with immune score. (C) PFS differences of stage T3 and T4 patients with and without radiation therapy. (D) PFS differences of patients classified by radiation therapy combined with immune score. (E) PFS differences between patients with or without lymph node metastasis. N− and N+, respectively, represents lymph node metastasis negative and positive. (F) PFS differences of patients classified by lymph node metastasis combined with immune score. (G) PFS differences between patients with high and low gleason scores. (H) PFS differences of patients classified by gleason score combined with immune score

There was no significant difference in PFS amongst men with advanced PC (stage T3 and stage T4) who received hormone therapy or not (*p* = 0.798, Figure 4A). The PC patients were grouped by IS combined with hormone therapy, and there were intergroup differences in PFS (*p* = 0.020). The PC patients with low IS and receiving hormone therapy had better prognosis than other groups (Figure 4B). There was no significant difference in PFS amongst men with advanced PC (stage T3 and T4) who received radiation therapy or not (*p* = 0.563, Figure 4C). The PC patients were grouped by IS combined with radiation therapy, but there were no intergroup differences in PFS (*p* = 0.793, Figure 4D). PC patients with a low gleason score (≤7) had better prognosis than those with a high gleason score (>7) (*p* = 0.005, Figure 4E). The PC patients were grouped by IS combined with gleason score. There were intergroup differences in PFS (*p* < 0.001). The PC patients with high IS and high gleason score had worse prognosis than other groups (Figure 4F). The PC patients without lymph node metastasis had a better prognosis than those with lymph node metastasis (*p* = 0.012, Figure 4G). The PC patients were grouped by IS combined with lymph node metastasis. There were intergroup differences in PFS (*p* < 0.001). The PC patients with high IS and lymph node metastasis had worse prognosis than other groups (Figure 4H). PC patients with lymph node metastasis had high IS than those with no lymph node metastasis (*p* = 0.002, Figure S1). There was no significant difference in the probability of lymph node metastasis between the high and low score groups (Chi^2^ = 2.268, *p* = 0.132, Figure S2).

## 4 DISCUSSION

Tumour tissue consists of tumour cells, immune cells, fibroblasts and cytokines. These elements interact to form the tumour microenvironment, which is closely correlated with the occurrence, progression and metastasis of cancers.^28^, ^29^, ^30^ Tumour cells can evade immune surveillance and establish an immunosuppressive environment through a variety of mechanisms.^31^, ^32^, ^33^, ^34^ Immunotherapy targeting immune checkpoints has been widely recognized as a treatment strategy for cancers. ICI has been shown to be correlated with clinical characteristics, and clusters based on ICI have been shown to be beneficial for determining therapeutic strategies.

Immunohistochemistry and flow cytometry have been conducted to explore ICI in previous studies; however, these strategies have limitations.^35^ Immunohistochemistry relies on cellular protein markers to identify immune cell subsets; however, this technique can only recognize a few of these subsets, and, because markers are also present in other types of cells, results can be biased.^36^, ^37^ Flow cytometry also depends on various protein markers to identify immune cell subtypes; however, this technique is limited by fluorescence channels.^38^, ^39^ For these reasons, most previous studies have been limited in their ability to determine the influence of one or several specific immune cell subtypes on the occurrence, progression, prognosis and treatment of tumours. Moreover, there has been a lack of comprehensive studies on the landscape of immune cells in tumour tissues, as well as the systematic characterization of immune cell subtypes that can be used to predict clinical outcomes, distinguish tumour heterogeneity and select treatment strategies.

Single sample gene set enrichment analysis is an excellent way to overcome these limitations. ssGSEA is a sophisticated deconvolution algorithm based on non‐overlapping genomes of specific immune cell subpopulations.^40^ The algorithm uses 783 specific immune cell‐based markers for prognostic assessments and treatment strategy selection. This algorithm has been validated by fluorescence‐activated cell sorting and has been applied to many types of tumours.^41^, ^42^

There was no comprehensive classification of the tumour immune microenvironment in PC. Some studies also attempted to provide answers from different perspectives.^43^, ^44^, ^45^, ^46^, ^47^ Combined with the components of immune invasion and characteristics of inflammatory response, tumour immune microenvironment of solid tumours can be roughly divided into immunosensitive and immunosuppressive.^44^ This is a standardized indicator for evaluating tumour immune environment based on T cells density and location. We provided another idea for clustering to characterize the immune microenvironment. We made more comprehensive use of information on the abundance of ICI, including 28 immune cell subtypes, not limited to T cells. In the selection of classification algorithm, we chose the NMF algorithm that can combine the abundance of ICI with the prognosis of PC patients. We used NMF algorithm to divide patients into two clusters, one with a better prognosis and higher levels of immune cell infiltration and the other with the opposite. Other classification methods, such as consensus clustering and K‐means, have also been applied to study tumour heterogeneity. Each of these models has its own characteristics. The most important feature of NMF is that the survival information of patients can be included, and the integrity of data information can be maintained. We confirmed the idea that high abundance of ICI predicted good prognosis. The expression levels of CTAL‐4 and PD‐L1 were lower in clusterB than in clusterA. This suggested that prostate cancer patients with high abundance of ICI may benefit more from immunotherapy. The infiltrative abundances, NMF‐cluster typing and prognostic value of immune cell subtypes in PC were confirmed. The abundance of 28 immune cell subtypes in each sample is a characteristic factor of the immune microenvironment, which can be used to characterize intratumour immune heterogeneity. This clustering may clarify the correlations between these immune cells subsets and prognosis of tumours in PC and provide reference for the selection of immunotherapy.

In order to explore the driving factors of immune microenvironment heterogeneity in PC, hub genes co‐expressed with NMF‐cluster typing were screened by WGCNA. These genes provided the basis for a much simpler algorithm that we developed to characterize intratumour immune heterogeneity later. Besides, the identification of these genes may reveal new information about the abundance of ICI and recruitment mechanism changes during tumour progression. CXCL10, CXCL5, MMP1, CXCL12, CXCL11, CXCL2, STAT1, IL‐6 and TLR2 were hub genes in network and may promote or inhibit the homing or of immune cells in tumours and immune cell differentiation, bring intratumoral immune heterogeneity. Previous studies have elucidated the role of these genes in tumours. IL‐6 regulates a variety of cellular functions, including cell proliferation, cell differentiation and haematopoiesis. In prostate cancer, IL‐6 can form autocrine/paracrine rings locally.^48^, ^49^ Paracrine action of IL‐6 inhibits the proliferation and induces cell differentiation of prostate cancer cells, whilst autocrine action stimulates the growth of prostate cancer cells.^49^ In addition to its direct effect on tumours, IL‐6 may regulate the immune microenvironment of prostate cancer by enriching immune cells. In combination with colony‐stimulating factor, it can promote the growth and differentiation of primitive bone marrow cells, enhance the lysis function of natural killer cells and the killing effect of effector T cells.^48^ MMPs is a class of endopeptidase, which can degrade extracellular matrix and protein components and destroy the interstitial barrier, and its increased expression level is conducive to the metastasis and invasion of tumour cells. In prostate cancer, MMP can promote tumour proliferation, invasion and metastasis and promote tumour progression.^50^, ^51^ MMP1 expression was positively correlated with the aggressiveness of prostate cancer subsets.^50^, ^51^ Multiple genes influence the biological behaviour of prostate cancer cells by regulating MMP1.^52^, ^53^, ^54^ STAT1 may activate the immune system of cancer patients.^55^, ^56^ However, abnormal low expression of STAT1 may create conditions for tumour immune escape.^57^ At present, some studies have confirmed that some kinds of cancer cells, including melanoma, squamous cell carcinoma and gastric carcinoma, can achieve the purpose of immune escape by downregulation of STAT1 expression.^58^, ^59^, ^60^ In prostate cancer, epigenetic changes of the STAT1 gene promoter and impairment of STAT1‐related signal transduction lead to impaired antigen presentation.^61^, ^62^, ^63^ However, it should be noted that the mechanism and effect of STAT1 are complex. STAT1 also inhibits the immune system. STAT1 can block T cell activation and promote T cell apoptosis by regulating arginase or some other enzymes. This phenomenon has been shown in breast cancer.^64^ In prostate cancer, CXCL family is a well‐known chemokine, whose main role is to induce the directional migration of immune cells.^61^, ^65^ The migration of immune cells attracted by chemokines to the chemokine source following the signal of increased chemokine concentration. These chemokines themselves also affect the biological behaviours of prostate cancer cells such as proliferation, differentiation and invasion.^66^, ^67^ Further studies are needed to confirm the regulatory role of these genes on immune cells during prostatic development and progression.

We performed dimensional reduction analysis to obtain IS based on the expression of hub genes via PCA. Immune score = ∑PC1(B+)–∑PC1(B−). ClusterB was poor in immune cells. So immune score may be negatively correlated to immune‐related genes. Immune‐related genes showed decreased expression in tumour samples with high immune score. We also found that high IS was companied with poor PFS and low immune checkpoint gene expression. We should note that there seemed to exist a positive correlation between genes that promote tumour immune interaction and immune checkpoint genes. Our analysis suggested that most immune checkpoint genes were expressed on immune cells. Immune checkpoint genes expression was positively correlated with immune cells infiltration. Immune checkpoint genes may be positively correlated with genes, which promote immune cell infiltration. Some studies showed the same trend. In the study of Zhang et al., the expression of immune checkpoint genes showed a consistent change trend with genes that promote tumour immune infiltration.^68^ Another study showed that PD‐1 expression was positively correlated with CD8 T cells.^69^ Of course, the specific relationship between the two needed further exploration and experimental confirmation. IS had good efficacy in predicting patient prognosis and immunotherapy sensitivity. The prognosis of patients with low IS receiving hormone therapy was significantly better than that of the other groups. Patients with advanced prostate cancer who had low IS were more sensitive to hormone therapy. However, we did not see similar results with radiation therapy. IS can provide a reference for prostate cancer patients to choose hormone therapy. IS combined with clinical features, including gleason score and lymph node metastasis, may accurately and finely predict the prognosis of patients. IS can further characterize the prognosis of high gleason score patients, which is generally considered to predict a poor prognosis. The combination of IS and lymph node metastasis can better differentiate patient outcomes than using it alone.

Of course, there were some flaws in our study. First, we lack in vivo and in vitro experiments to verify our results, which was the biggest drawback of our study; Secondly, we failed to clarify the specific mechanism of action of hub gene. Finally, IS can be combined with a wider range of clinical factors to provide a reference for patient treatment selection and prognosis assessment. We planned to carry out a continuation study to perfect these defects in experiments.

In conclusion, NMF clustering based on the infiltrating abundance of immune cell subtypes can distinguish prognosis of prostate cancer patients, predict immunotherapy sensitivity and depict intratumoral immune heterogeneity. IS was a practical algorithm to predict the prognosis of patients. Advanced prostate cancer patients with low IS may be more sensitive to hormone therapy. CXCL10, CXCL5, MMP1, CXCL12, CXCL11, CXCL2, STAT1, IL‐6 and TLR2 were hub genes, which may drive the homing of immune cells in tumours and promote immune cell differentiation.

## AUTHOR CONTRIBUTIONS

**jianpeng han:** Data curation (lead); formal analysis (lead); investigation (lead); methodology (lead); software (lead); validation (lead); visualization (lead); writing – original draft (lead); writing – review and editing (lead). **yan zhou:** Data curation (supporting); formal analysis (supporting); investigation (supporting); software (supporting); writing – original draft (supporting). **chundong zhang:** Data curation (supporting); writing – original draft (supporting); writing – review and editing (supporting). **jianyong feng:** Data curation (supporting); writing – original draft (supporting); writing – review and editing (supporting). **junhao wang:** Data curation (supporting); writing – original draft (supporting); writing – review and editing (supporting). **kuo guo:** Data curation (supporting); writing – original draft (supporting); writing – review and editing (supporting). **wenbin chen:** Formal analysis (supporting); writing – original draft (supporting); writing – review and editing (supporting).

## FUNDING INFORMATION

This work was supported by Fund of Hebei Provincial Health Commission, No: 20210302. Internal fund of Hebei University of traditional Chinese medicine, No: KTY2019015. Project of Hebei administration of traditional Chinese medicine, No: 2022040.

## CONFLICT OF INTEREST

The authors declare that they have no competing interests.

## Supporting information


FigureS1

FigureS2

TableS1

## Data Availability

The datasets used and/or analysed during the current study are available from the corresponding author on reasonable request.
