# Supplementary figures and images for: Intratumoral immune heterogeneity of prostate cancer characterized by typing and hub genes
Source: J Cell Mol Med. 2022 Dec 16;27(1):101–12. doi: 10.1111/jcmm.17641 (PMC9806298; doi:10.1111/jcmm.17641)

Lymphnode metastasis    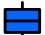 No metastasis    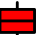 Metastasis

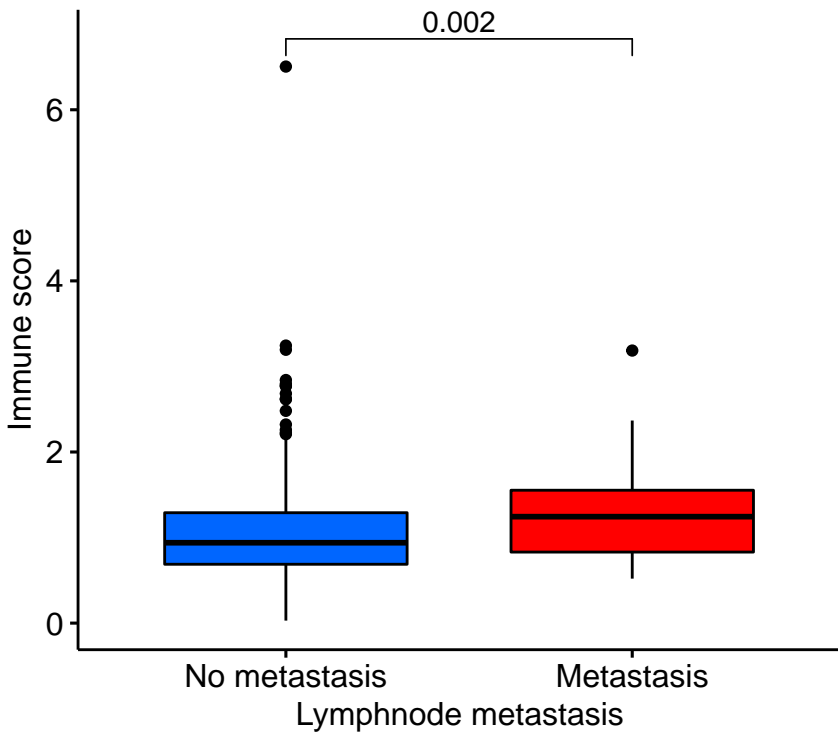

Supplement: Supplementary file 1 — FigureS1 [file JCMM-27-101-s001.pdf]

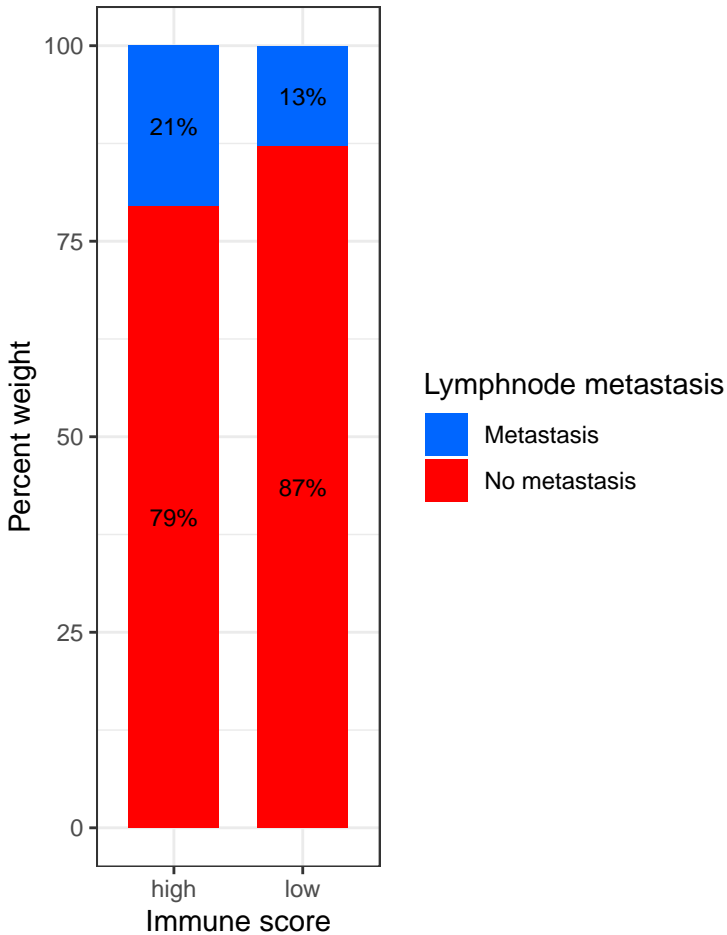

Supplement: Supplementary file 2 — FigureS2 [file JCMM-27-101-s002.pdf]
